# On a generalized magnitude system in the brain: an integrative perspective

**DOI:** 10.3389/fpsyg.2013.00829

**Published:** 2013-11-12

**Authors:** Carmelo M. Vicario

**Affiliations:** School of Psychology, The University of QueenslandBrisbane, QLD, Australia

**Keywords:** time, space, numbers, perception, cognition

Research in the field of cognitive neuroscience has provided extensive evidence in support of a strong relationship between time, space, and number in the human brain. In 1993, Dehaene et al. discovered the SNARC (spatial-numerical association of response codes) effect and suggested that numbers may be internally represented along a spatial dimension, with low numbers on the left of a mental number line, and high numbers on the right. The investigation of this effect has been studied through the use of different methodologies and recent insights have extent this effect also to decisional processes (Daar and Pratt, 2008; Loetscher et al., 2008; Vicario, 2012, 2013).

Similar relationships were also documented in research investigating link between space and time (DeLong, 1981; Vicario et al., 2007, 2009, 2011a,b; Ishihara et al., 2008; Vallesi et al., 2008; Fabbri et al., 2013) as well as between time and numbers (for instance see Dormal et al., 2006; Vicario et al., 2008; Lu et al., 2009; Vicario, 2011).

This literature corroborates the suggestion of common cortical metrics for space time and numbers (Walsh, 2003). However, despite the 10 years that have elapsed since Walsh (2003) published his theoretical work, the debate about “A theory of Magnitude” (ATOM) is still object of discussion.

This research topic entitled “On a generalized magnitude system in the brain: insights from experimental evidence” contains 10 state-of-the-art original research articles exploring the relationships between time, space and number. These research articles provide original contributions about the representation of magnitude in different research fields including childhood participants (Bottini and Casasanto, 2013; Lonnemann et al., 2013; Tokita and Ishiguchi, 2013), genetic polymorphism (Júlio-Costa et al., 2013), clinical populations such as schizophrenia (Martinez-Cascales et al., 2013) and right brain damaged patients (Ishihara et al., 2013), investigations on healthy adults (Baker et al., 2013; Blini et al., 2013; Crollen et al., 2013; Viarouge and de Hevia, 2013).

A lively contribute to this debate is also provided by the mini-review of Leibovich and Henik (2013) and six opinion articles (Agrillo and Miletto Petrazzini, 2013; Arzy et al., 2013; De Simone, 2013; Tzelgov et al., 2013; Van Opstal and Verguts, 2013; Vicario et al., 2013). These theoretical contributions address ATOM from different perspectives, revealing the strengths but also the weaknesses of this model.

Much more work needs to be done and many issues remain to be addressed before we fully understand the brain mechanisms underlying the existence of analogies (Vicario and Martino, 2010) between magnitudes.

It has been a great pleasure and honor to be involved in this Research Topic. I would like to thank all of the authors, reviewers, and Frontiers staff for helping to make this Research Topic possible and I look forward to further explorations of the brain mechanisms underlying ATOM.

## References

[B1] Agrillo C. Miletto Petrazzini M. E. (2013). Glimpse of ATOM in non-human species. Front. Psychol. 4:460 10.3389/fpsyg.2013.00460 23888148 PMC3719011

[B2] Arzy S. (2013). When speaking of the experience, do not leave out the experiencer: on self and magnitude. Front. Psychol. 4:303 10.3389/fpsyg.2013.00303 23755032 PMC3664762

[B3] Baker J. M. Rodzon K. S. Jordan K. (2013). The impact of emotion on numerosity estimation. Front. Psychol. 4:521 10.3389/fpsyg.2013.00521 23950754 PMC3739062

[B4] Blini E. Cattaneo Z. Vallar G. (2013). Different effects of numerical magnitude on visual and proprioceptive reference frames. Front. Psychol. 4:190 10.3389/fpsyg.2013.00190 23616777 PMC3627981

[B5] Bottini R. Casasanto D. (2013). Space and time in the child's mind: metaphoric or ATOMic? Front. Psychol. 4:803 10.3389/fpsyg.2013.00803 PMC3817359 24204352

[B6] Crollen V. Grade S. Pesenti M. Dormal V. (2013). A common metric magnitude system for the perception and production of numerosity, length, and duration. Front. Psychol. 4:449 10.3389/fpsyg.2013.00449 23885244 PMC3717486

[B7] Daar M. Pratt J. (2008). Digits affect actions: the SNARC effect and response selection. Cortex. 44, 400–405 10.1016/j.cortex.2007.12.003 18387571

[B8] Dehaene S. Bossini S. Giraux P. (1993). The mental representation of parity and numerical magnitude. J. Exp. Psychol. Gen. 122, 371–396

[B9] DeLong A. J. (1981). Phenomenological space-time: toward an experiential relativity. Science 213, 681–683 10.1126/science.7256273 7256273

[B10] De Simone L. (2013). Grounding magnitudes. Front. Psychol. 4:410 10.3389/fpsyg.2013.00410 23847578 PMC3703566

[B11] Dormal V. Seron X. Pesenti M. (2006). Numerosity-duration interference: a stroop experiment. Acta Psychol. 121, 109–124 10.1016/j.actpsy.2005.06.003 16095549

[B12] Fabbri M. Cellini N. Martoni M. Tonetti L. Natale V. (2013). Perceptual and motor congruency effects in time-space association. Atten. Percept. Psychophys. [Epub ahead of print]. 10.3758/s13414-013-0519-9 23904320

[B13] Ishihara M. Keller P. E. Rossetti Y. Prinz W. (2008). Horizontal spatial representations of time: evidence for the STEARC effect. Cortex 44, 454–461 10.1016/j.cortex.2007.08.010 18387578

[B14] Ishihara M. Revol P. Jacquin-Courtois S. Mayet R. Rode G. Boisson D. (2013). Tonal cues modulate line bisection performance: preliminary evidence for a new rehabilitation prospect. Front. Psychol. 4:704 10.3389/fpsyg.2013.00704 24109467 PMC3791388

[B15] Júlio-Costa A. Antunes A. M. Lopes-Silva J. B. Moreira B. C. Vianna G. S. Wood G. (2013). Count on dopamine: influences of COMT polymorphisms on numerical cognition. Front. Psychol. 4:531 10.3389/fpsyg.2013.00531 23966969 PMC3744013

[B16] Leibovich T. Henik A. (2013). Magnitude processing in non-symbolic stimuli. Front. Psychol. 4:375 10.3389/fpsyg.2013.00375 23805121 PMC3691512

[B17] Loetscher T. Schwarz U. Schubiger M. Brugger P. (2008). Head turns bias the brain's internal random generator. Curr. Biol. 18, R60–R62 10.1016/j.cub.2007.11.015 18211838

[B18] Lonnemann J. Linkersdörfer J. Nagler T. Hasselhorn M. Lindberg S. (2013). Spatial representations of numbers and letters in children. Front. Psychol. 4:544 10.3389/fpsyg.2013.00544 23970877 PMC3748714

[B19] Lu A. Hodges B. Zhang J. Zhang J. X. (2009). Contextual effects on number-time interaction. Cognition 113, 117–122 10.1016/j.cognition.2009.07.001 19640515

[B20] Martinez-Cascales I. De La Fuente J. Santiago J. Santiago J. (2013). Space and time bisection in schizophrenia. Front. Psychol. 4:823 10.3389/fpsyg.2013.00823 PMC3817397 24204358

[B21] Tokita M. Ishiguchi A. (2013). Effects of perceptual variables on numerosity comparison in 5-6-year-olds and adults. Front. Psychol. 4:431 10.3389/fpsyg.2013.00431 23898308 PMC3721045

[B22] Tzelgov J. Zohar-Shai B. Nuerk H. C. (2013). On defining quantifying and measuring the SNARC effect. Front. Psychol. 4:302 10.3389/fpsyg.2013.00302 23750147 PMC3667495

[B23] Vallesi A. Binns M. A. Shallice T. (2008). An effect of spatial–temporal association of response codes: understanding the cognitive representations of time. Cognition 107, 501–527 10.1016/j.cognition.2007.10.011 18076872

[B24] Van Opstal F. Verguts T. (2013). Is there a generalized magnitude system in the brain. Behavioral, neuroimaging, and computational evidence. Front. Psychol. 4:435 10.3389/fpsyg.2013.00435 23874319 PMC3711208

[B25] Viarouge A. de Hevia M. D. (2013). The role of numerical magnitude and order in the illusory perception of size and brightness. Front. Psychol. 4:484 10.3389/fpsyg.2013.00484 23908640 PMC3725837

[B26] Vicario C. M. (2011). Perceiving numbers affects the subjective temporal midpoint. Perception 40, 23–29 10.1068/p6800 21513181

[B27] Vicario C. M. (2012). Perceiving numbers affects the internal random movements generator. Sci. World J. 2012:347068 10.1100/2012/347068 22629133 PMC3353301

[B28] Vicario C. M. (2013). Landmark test and decision making: a reply to a reply. Perception 42, 356–357 10.1068/p7334 23837211

[B29] Vicario C. M. Bonní S. Koch G. (2011a). Left hand dominance affects supra-second time processing. Front. Integr. Neurosci. 5:65 10.3389/fnint.2011.00065 22028685 PMC3199548

[B30] Vicario C. M. Martino D. Pavone E. F. Fuggetta G. (2011b). Lateral head turning affects temporal memory. Percept. Mot. Skills 113, 3–10 10.2466/04.22.PMS.113.4.3-10 21987905

[B31] Vicario C. M. Caltagirone C. Oliveri M. (2007). Optokinetic stimulation affects temporal estimation in healthy humans. Brain Cogn. 64, 68–73 10.1016/j.bandc.2006.12.002 17397979

[B32] Vicario C. M. Martino D. (2010). The neurophysiology of magnitude: one example of extraction analogies. Cogn. Neurosci. 1, 144–145 10.1080/17588921003763969 24168285

[B33] Vicario C. M. Pecoraro P. Turriziani P. Koch G. Caltagirone C. Oliveri M. (2008). Relativistic compression and expansion of experiential time in the left and right space. PLoS ON. 3:e1716 10.1371/journal.pone.0001716 18320037 PMC2248621

[B34] Vicario C. M. Rappo G. Pepi A. Oliveri M. (2009). Timing flickers across sensory modalities. Perception 38, 1144–1151 10.1068/p6362 19817148

[B35] Vicario C. M. Yates M. J. Nicholls M. E. (2013). Shared deficits in space, time, and quantity processing in childhood genetic disorders. Front. Psychol. 4:43 10.3389/fpsyg.2013.00043 23405055 PMC3566548

[B36] Walsh V. (2003). A theory of magnitude: common cortical metrics of time, space and quantity. Trends Cogn. Sci. 11, 483–488 10.1016/j.tics.2003.09.002 14585444

